# The Book World of Medicine and Science

**Published:** 1906-10-13

**Authors:** 


### The Book World of Medicine and Science.

Second Report of the Wellcome Research Laboratories at the Gordon Memorial College, Khartoum. By Andrew Balfour, M.D., B.Sc., F.R.C.P.Edin., D.P.H.Camb., Director, Department of Education, Sudan Government, Khartoum. 1906.

The second report of the Wellcome Research Laboratories at the Gordon Memorial College, Khartoum, has recently appeared. It is brought out and edited by Dr. Balfour, the Director of the institution, and forms a compendium of the year's work accomplished there. The first report appeared in 1904, and since that date the author states that the record has been one of steady progress, with a great increase in the amount and variety of the work, additions to the staff having rendered it possible to cope with the routine duties, while some efforts at research have also been made. This latter sentence sums up the work contained in the report, the bulk of the contained matter consisting of classification of species of flies, routine work on the prophylaxis of malaria, with research work on trypanosomiasis, and other protozoal parasites of mammals, reptiles, birds, and fishes, subjects which are now of supreme importance.

The classification of the various mosquitoes and flies discovered in the Sudan by the various workers has been entrusted to the hands of Messrs. Austen and Theobald, in England. Those experts have each written chapters in the report in which there is little to criticise. The former draws attention to the ignorance which prevails amongst doctors and others as to how to collect and properly pack insects for sending home to England. This is a branch which must be cultivated in the future, as inattention to a few preliminary rules leads to a great loss of time and loss of material; for example, on p. 202 Dr. Sheffield Neave, the travelling pathologist, writes: "A collection was made (insects), but this was more than half destroyed in its transit to England." Now why should this collection have been destroyed? Presumably because they were not properly mounted and packed in the first instance, and not properly secured in boxes for their long journey. The anti-mosquito work in Khartoum and the Anglo-Egyptian Sudan generally may just be mentioned in passing; there is nothing new in it, but its results are hopeful. Dr. Balfour on p. 26 states: "Therefore, for something considerably under £100 per annum, Khartoum is kept practically free from malaria, and the inhabitants are secured, to a very great extent, from the persistent and annoying attentions of those winged pests, which, as a rule, add so much discomfort to life in the tropics. I do not think the above is a large sum to pay for such immunity." With this we heartily agree, and such a statement shows how much can be done, if the place is favourable, by a little common sense. Turning now to the scientific work, we find a very interesting paper entitled "A Hæmogregarine of Mammals," by Dr. Balfour. The discovery of such parasites in the red blood corpuscles of mammals is quite new to science, and the discoverer must be heartily congratulated on his find. The work on the possible extracorporeal cycle of this interesting parasite is not quite convincing, perhaps, but investigations into the development of parasites in minute insects are of extreme difficulty, with pitfalls around in every direction to entrap the unwary. The commencement of his work on insects illustrates this forcibly—namely, the finding of the *Crithidia* in fleas. The work of Ross on the intestinal parasites of mosquitoes, and Novy and McNeal's work on trypanosomes, tend to show that an authority of the weight of Schaudinn even has fallen into one of the pitfalls before mentioned. The chapter on Trypanosomasis in the Anglo-Egyptian Sudan, though useful, forms rather uninteresting reading, the repetition of so many inoculation experiments becoming wearisome, and then after all no very definite conclusions, apparently, are reached from them. Dr. Neave's part of the work, entitled the "Report of Travelling Pathologist and Naturalist," illustrates the difficulties of what may be termed "bush" pathology, and also the difficulties of having a committee constantly meddling with and altering the individual's plan of action. A perusal of p. 183 explains what is meant; fancy arriving at one's headquarters and having to wait twenty-three days for one's outfit. Again Dr. Neave was expected to accomplish too much in the limited time at his disposal, and such being the case his work suffers accordingly. He seemed to be constantly on the rush, and perhaps if he had limited himself to the catching of tsetse flies alone it would have been better. Some of his work—for example, the new filariæ found in birds—loses much of its value because time was not apparently available for careful dissections to be made in order to find the adult forms. Without those, definite classification is impossible. Dr. Beam's report of the chemical section is very interesting and instructive, and should prove most useful to the Sudan Government. Before concluding one must draw attention to the very excellent coloured plates and other diagrams profusely scattered throughout the text. Mr. Terzi's illustrations of the flies are extremely accurate, beautifully drawn, and should form standards which it will be very difficult ever to surpass. They contribute to the value of the report enormously. Mr. Richard Muir's delineations of the blood parasites are also excellent, while the microphotographs and photographs are all nicely reproduced. No expense apparently has been spared in the production of the report, the binding, the paper, the drawings are all of the best, and one can hardly recollect, even at this day when reports are so common, such a marvellously got up work. This, in addition of course to the value of the work contained within its pages, must be very pleasing to Dr. Balfour, the editor, and he is to be congratulated on his success.

